# Heterogeneity of Renal Endothelial Cells, Interact with Neighboring Cells, and Endothelial Injury in Chronic Kidney Disease: Mechanisms and Therapeutic Implications

**DOI:** 10.7150/ijms.108299

**Published:** 2025-04-09

**Authors:** Meiyu Zhang, Wu Liu, Haoran Dai, Hanxue Jiang, Qihan Zhao, Wenbin Liu, Hongliang Rui, Baoli Liu

**Affiliations:** 1 Beijing Hospital of Traditional Chinese Medicine, Capital Medical University, Beijing 100010, China.; 2 Beijing University of Chinese Medicine, Beijing 100029, China.; 3 Shunyi Branch, Beijing Hospital of Traditional Chinese Medicine, Capital Medical University, Beijing 100310, China.; 4 Laboratory for Clinical Medicine, Capital Medical University, Beijing 100069, China.; 5 Beijing Research Institute of Chinese Medicine, Beijing University of Chinese Medicine, Beijing 100029, China.

**Keywords:** Chronic Kidney Disease, Endothelial Heterogeneity, Endothelial dysfunction, Endothelial injury

## Abstract

Chronic kidney disease (CKD) is closely associated with endothelial dysfunction, leading to symptoms such as albuminuria, edema, and coagulopathy. Recent advancements in single-cell sequencing have deepened our understanding of the heterogeneity of renal endothelial cells, which is significantly influenced by their microenvironment. Understanding the influence of neighboring cells on endothelial heterogeneity is essential for elucidating the mechanisms underlying vascular dysfunction and CKD progression. This review explores the latest research on renal endothelial cell heterogeneity and their interactions with neighboring cells. We further discuss the mechanisms of endothelial injury in CKD, including alterations to the endothelial glycocalyx, inflammation, oxidative stress, and dysfunction of the glomerular filtration barrier. Renal endothelial injury contributes to complications, including cardiovascular disease, diabetic nephropathy, and impaired vascular function. Therapeutic strategies encompass antihypertensive, hypoglycemic, and lipid-lowering treatments, supplemented by emerging approaches such as anti-inflammatory therapies, gene therapy, and lifestyle modifications. Through reviewing the relationship between endothelial injury and CKD progression, we emphasize potential strategies to enhance prognosis and mitigate disease progression.

## 1. Introduction

Endothelial cells (ECs), also known as vascular endothelial cells, form a monolayer lining the inner surfaces of arteries, veins, and capillaries. These cells have a luminal membrane exposed to blood and circulating cells, while their basolateral surface is supported by a glycoprotein basement membrane that they produce^1^. ECs play critical roles in barrier functions, filtration, angiogenesis, and the regulation of vascular tone^2^. Additionally, they are involved in immune responses, inflammation, and maintaining the balance between coagulation and fibrinolysis^3^,^4^. Recent research has provided detailed insights into the roles of vascular endothelium in mechanotransduction, metabolism, guidance signaling, and aging^5^. Therefore, studying ECs is essential for understanding various pathological conditions.

Recent studies have identified distinct subsets of ECs, including glomerular ECs and those forming the blood-brain barrier^6^. Advances in technology, particularly single-cell RNA sequencing (scRNA-seq), have significantly deepened our understanding of these subsets. Compared to traditional methods such as serial analysis of gene expression, microarrays, or bulk RNA sequencing, scRNA-seq enables a more comprehensive analysis by integrating transcriptomic profiles across diverse cell types^7^-^9^.

CKD affects approximately 10% of the global adult population and is becoming increasingly prevalent^10^. CKD arises from a variety of pathological conditions, influenced by factors such as blood glucose, lipid levels, homocysteine, and bilirubin, which impact tissues through the circulatory system. These changes alter the microenvironment within tissues. Clinically, CKD is characterized by symptoms such as edema, proteinuria, coagulation abnormalities, and a reduced glomerular filtration rate (GFR), all of which are associated with endothelial cell damage^11^,^12^.

Recent research has delved into these complexities. Sedrakyan et al. classified renal endothelial cells based on transcriptomic differences and reviewed several scRNA-seq studies that explored the effects of CKD and acute kidney injury (AKI) on the renal endothelium^13^. Despite these advancements, the underlying causes of endothelial heterogeneity and the mechanisms of injury remain poorly understood. This study seeks to unravel the origins of renal endothelial cell heterogeneity by examining intra-tissue cell interactions. Furthermore, it consolidates current insights into the mechanisms and clinical manifestations of endothelial cell damage in CKD, offering new perspectives for identifying therapeutic targets related to the interplay between endothelial cells and CKD.

## 2. Heterogeneity of Renal Endothelial Cells

The renal vascular system exhibits remarkable diversity, with blood entering the kidney via the renal artery, passing through the glomerular capillaries, and exiting through the efferent arterioles. The renal vasculature adapts to distinct microenvironments, demonstrating specialization in endothelial cell structure, blood flow dynamics, and fenestration. Early studies employing electron microscopy and microarray analysis highlighted significant heterogeneity in the morphology and function of renal endothelial cells across various vascular sites. Techniques such as immunostaining and FACS analysis have identified renal endothelial markers, including Erg, VE-cadherin, Meca32, Thrombomodulin, and vWF^1^, yet further classification of renal endothelium remains necessary^14^.

### 2.1 Endothelial Cell Subtypes and Characteristics

Advances in scRNA-seq technology have significantly enhanced our understanding of individual cell types, uncovering organ-specific gene expression signatures^9^. For instance, Jihwan Park et al. constructed a single-cell transcriptome atlas of mouse kidneys, producing the most comprehensive map of kidney transcriptomes to date^15^. Their findings suggest that these data can facilitate the inference of cell type-specific functions and link numerous genetic kidney diseases to specific cell types.

Dumas et al. employed scRNA-seq to comprehensively characterize the transcriptional landscape of kidney endothelial cells in adult mice, identifying 24 transcriptionally distinct subtypes, including 5 glomerular, 9 cortical, and 10 medullary EC subtypes^7^. Within the cortical subtypes, specific populations such as large artery endothelial cells, afferent arterioles, efferent arterioles, four distinct capillary subtypes, and large vein endothelial cells were delineated. Furthermore, Dumas et al. highlighted gene-specific markers associated with endothelial cell function and their adaptation to the local microenvironment. For example, in glomerular endothelial subtypes, genes such as *Edn1*, *Alox12*, and *S1pr1*, which are implicated in the regulation of angiotensin signaling, were found to be selectively expressed in afferent arterioles. Notably, the S1P-S1PR1 signaling pathway was shown to regulate angiotensin levels by activating the nitric oxide synthase (eNOS) system, a critical mechanism for maintaining glomerular blood flow and preserving GFR^16^.

Several genes remain under-characterized. For example, the ELN gene, expressed in large artery endothelial cells, encodes elastin, a protein crucial for vascular elasticity and associated with renal cyst progression and diabetic nephropathy^17^,^18^. The Calca gene, expressed in efferent arterioles, encodes calcitonin, a hormone that influences G protein-coupled receptor signaling and NMDA receptor function, regulating vascular tone by modulating calcium influx^19^. Col4a1 and Col4a2, expressed in capillary endothelial cells, encode collagen type IV proteins in the basement membrane and are linked to vascular diseases. Studies have shown that the Col4a1 G498V mutation can delay glomerular development and podocyte differentiation, underscoring its role in kidney vascular and podocyte development^20^,^21^.

Furthermore, gene expressions are also connected to renal tubular function. For instance, the Jup gene, encoding Aquaporin-2 and expressed in the post-capillary venule subtype, interacts with the transcription regulator β-catenin and contributes to the renal anti-diuretic response^22^.

### 2.2 Involvement of Endothelial Cell Subtypes in Vascular Dysfunction and CKD Pathogenesis

It is well-established that different endothelial cell subtypes play critical roles in the progression of CKD by regulating vascular permeability, inflammatory responses, vascular tone, fibrotic signaling, and microvascular integrity. These subtypes exhibit unique characteristics and gene expression profiles, which collectively determine their central functions in disease mechanisms^23^.

Regulation of vascular permeability is a key function of endothelial cell subtypes. Capillary endothelial subtypes express crucial genes involved in permeability regulation, including VEGF and Plvap^24^,^25^. Aberrant expression of VEGF in glomerular collapse leads to rapid loss of glomerular endothelial cells (gRECs) and proteinuria, while PV1, the protein product of Plvap, facilitates water, ion, and solute exchange by covering endothelial fenestrae^26^. In the medullary capillary plexus, Plvap works in concert with VEGF receptor genes such as Kdr, Flt1, and Nrp1 to maintain vascular barrier function. However, in CKD, dysfunction of these genes disrupts the barrier, increasing inflammatory cell infiltration and tissue damage, thereby exacerbating inflammation, fibrosis, and renal dysfunction^16^.

Inflammatory responses are central to CKD progression, with specific endothelial cell subtypes exhibiting significant pro-inflammatory properties. For example, glomerular endothelial cells derived from efferent arterioles express Klf2, Klf4, and their target gene Thbd, which are suppressed under conditions of low shear stress, triggering pro-inflammatory signaling that worsens both local and systemic inflammation^27^. Studies have shown that activation of KLF2 protects gRECs from CKD-related injury^28^. Additionally, certain endothelial subtypes, such as capillary endothelial cells of the interferon (IFN) response phenotype, express Isg15 and Ifit gene families, which are involved in antigen processing and presentation, suggesting their potential role in CKD-associated inflammatory responses^29^.

Regulation of vascular tone depends on the secretion of vasoactive substances such as nitric oxide (NO) and endothelin-1 (ET-1) by endothelial subtypes. For instance, capillary endothelial cells express NOSTRIN, whose protein product interacts with eNOS to regulate NO production^30^,^31^. In CKD, dysfunction of NOSTRIN leads to reduced NO production, enhanced vasoconstriction, and decreased blood flow, exacerbating renal ischemia and injury^32^. Furthermore, eNOS uncoupling induces endothelial surface remodeling, promoting receptor expression and facilitating interactions with platelets and immune cells, thereby aggravating coagulopathy and disease progression^33^,^34^.

Fibrotic signaling and microvascular integrity are further disrupted during CKD progression. Certain capillary endothelial subtypes express genes such as Apln, Aplnr, Col4a1, Col4a2, Esm1, and Fscn1, which play essential roles in angiogenesis and fibrotic signaling. Additionally, venous endothelial cells of the IFN response phenotype express Isg15 and Ifit gene families, potentially contributing to immune regulation in fibrosis. The loss of microvascular barrier function creates a feedback loop that exacerbates inflammation and fibrosis^11^,^35^.

## 3. Interaction of Glomerular Endothelial Cells (GECs) with Neighboring Cells

Recent research has shown that the microenvironment significantly influences the development of endothelial cells in various tissues. In the kidney, the gene expression of renal endothelial cells is closely linked to signals from neighboring cells. GECs, key components of blood vessel walls, engage in complex communication with tubular epithelial cells, interstitial cells, and immune cells. This signaling network plays a critical role in regulating glomerular filtration, maintaining vascular tone, and modulating the inflammatory response in the glomerulus^36^ **(Table 1)**.

### 3.1 GECs Interact with Podocytes

GECs are connected to podocytes through the glomerular basement membrane (GBM) within the glomerular filtration barrier. Their differentiation is regulated by key signaling molecules, including vascular endothelial growth factor-A (VEGF-A), angiopoietin (Ang), and ET-1, which are secreted by podocytes.

In the kidney, podocyte-derived VEGF-A is essential for maintaining the structure and function of glomerular capillaries^37^. VEGF-A also protects podocytes from apoptosis by promoting nephrin phosphorylation and enhancing the podocin-CD2-associated protein (CD2AP) interaction^55^. Additionally, VEGF-C increases endothelial fenestration density, reduces albumin permeability, and lowers microalbuminuria in patients with diabetic kidney disease^36^,^38^. The specific knockout of the VEGF gene in mouse podocytes leads to endothelial abnormalities and thrombotic microangiopathy, highlighting the importance of VEGF in these processes^37^.

Ang, a key vascular growth factor involved in vascular remodeling and stability, is widely expressed in the kidney. Podocyte-derived Ang-1 interacts with the Tie-2 receptor on GECs, promoting endothelial survival. Tie-2 activation triggers Akt-dependent phosphorylation, inactivating forkhead box protein O1 (FOXO1) and suppressing gene expression linked to endothelial instability and apoptosis. This pathway supports vascular integrity, enhances cell survival, and promotes vascular stability. Ang-1 also exerts anti-inflammatory effects by inhibiting tumor necrosis factor-alpha (TNF-α)-induced leukocyte migration, suppressing damage-induced angiogenesis and fibrosis, and protecting glomerular capillaries from high blood sugar and other harmful factors^39^.

ET-1, a potent vasoconstrictor peptide secreted by endothelial cells, mediates communication between podocytes and GECs. Podocyte-derived ET-1 induces calcium influx in GECs, regulating vascular tone and glomerular hemodynamics. ET-1 also stimulates endothelial proliferation and cytokine production, influencing inflammation and fibrosis in GECs^40^. Additionally, endothelial-derived ET-1 regulates podocyte function and differentiation. By binding to podocyte receptors, ET-1 affects podocyte morphology, function, and proliferation, and regulates extracellular matrix synthesis and secretion. Activation of the ETAR on podocytes triggers the mitogen-activated protein kinase (MAPK), p21waf/cip1, and nuclear factor-kappa B (NF-κB) pathways, disrupting the F-actin cytoskeleton and impairing slit diaphragm function via Rho kinase and phosphoinositide 3-kinase (PI3 kinase) activation^41^.

### 3.2 GECs Interact with Renal Tubular Epithelium

The balance between glomerular-tubular interactions and feedback mechanisms is essential for maintaining renal metabolic function. GECs are closely linked with tubular epithelial cells, forming a complex network within the renal microenvironment.

Research has shown the significant role of the klotho protein in the kidney. Studies on gene-deficient mice reveal endothelial dysfunction, highlighting klotho's importance in renal homeostasis. Angiotensin-(1-7), a bioactive peptide produced by tubular epithelial cells, binds to the Mas receptor, activating the klotho and Nrf2/HO-1 pathways^42^,^43^. This mechanism helps inhibit GEC aging and preserves renal function. Additionally, tubular epithelial cells secrete VEGF, which binds to VEGFR on GECs, promoting endothelial differentiation and supporting renal microvasculature integrity^44^.

Endothelial cells also influence tubular epithelial cells by releasing NO and various growth factors and regulatory proteins. For instance, insulin-like growth factors (IGFs) expressed by GECs regulate renal cell growth and function, with IGFBPs modulating IGF signaling in tubular epithelial cells^45^.

### 3.3 GECs Interact with Glomerular Mesangial Cells (MCs)

The strategic location of MCs within the glomerulus positions them as a hub for intercellular communication^46^. Ang-1 and Ang-2 are thought to competitively regulate GEC proliferation and differentiation via the Tie-2 receptor^43^. In models of mesangial proliferative glomerulonephritis (MPGN), co-culture studies have shown that mesangial cell-derived VEGF-A induces the expression of VEGF receptor 2 and Ang-2 in GECs, inhibiting Tie-2 phosphorylation and modulating GEC proliferation^47^. Mesangial cells also influence GEC secretion of ET-1, as demonstrated in co-culture experiments, which show decreased mRNA and protein levels of endothelin-converting enzyme-1 (ECE-1)^48^.

Research indicates that MC development relies on endothelial cell-derived PDGF-B^49^. Endothelial cells also release NO, altering cGMP levels in MCs, thereby impacting their structure and function^50^. In vitro studies further reveal that extracellular vesicles from endothelial cells are internalized by MCs, promoting proliferation and matrix production via the TGF-β1/Smad3 pathway^46^,^51^.

### 3.4 GECs Interact with Glomerular Parietal Epithelium

**Figure 1:**
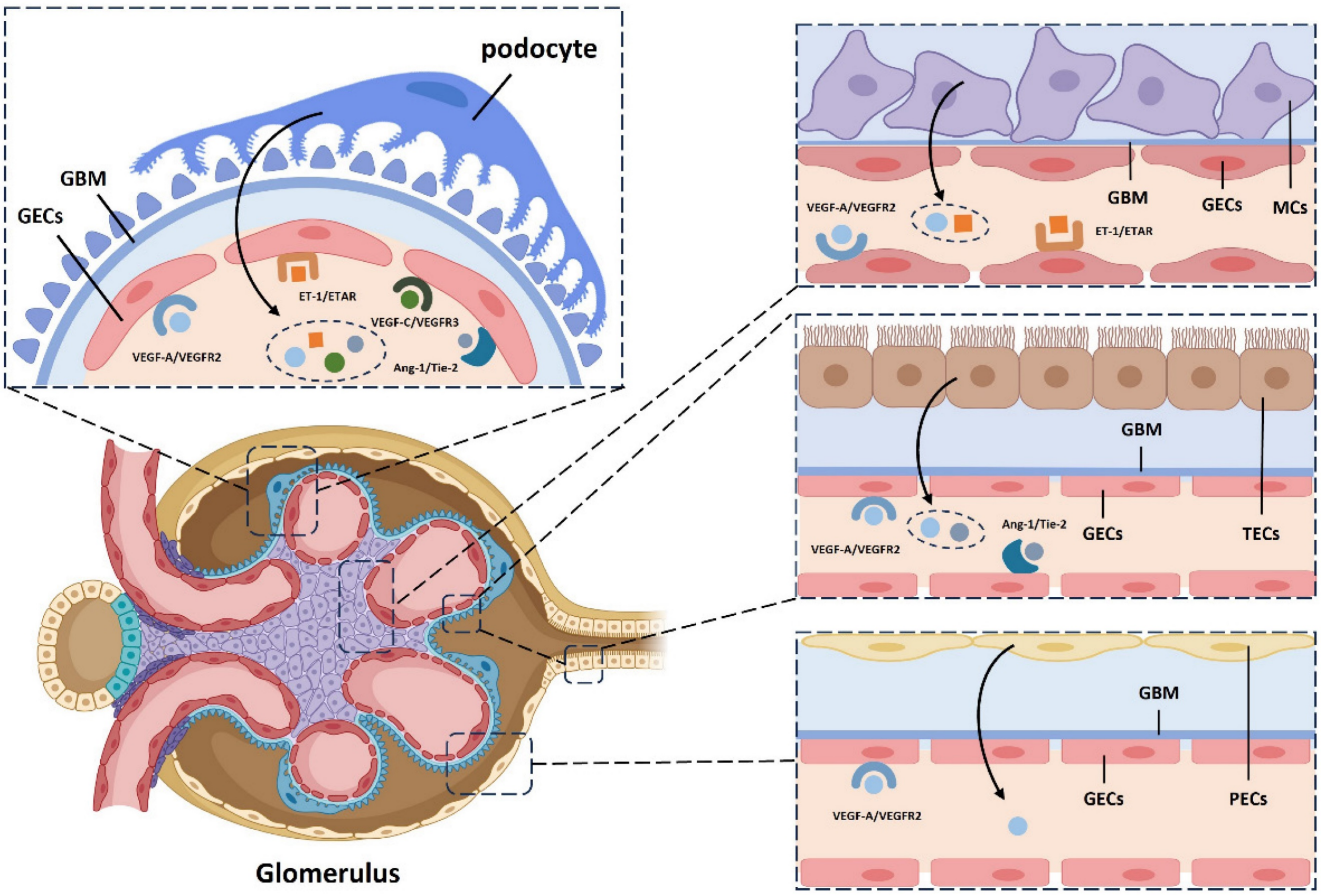
Enhanced schematic illustrating the influence of diverse cellular signaling pathways on the differentiation and development of glomerular endothelium. GBM, Glomerular Basement Membrane; GECs, Glomerular Endothelial Cells; TECs, Tubular Epithelial Cells; MCs, Mesangial Cells; PECs, Parietal Epithelial Cells; VEGF-A, Vascular Endothelial Growth Factor A; VEGFR2, Vascular Endothelial Growth Factor 2 Receptor; Ang-1, Angiopoietin-1; ET-1, Endothelin-1; ETAR, Endothelin A Receptor.

While the influence of the glomerular parietal epithelium on GECs is less prominent than that of other cell types, it still plays a role in modulating endothelial function. Glomerular epithelial cells, derived from mesenchymal cells, are located near GECs along the vascular lumen. Studies have shown that VEGF release by glomerular epithelial cells promotes fenestration formation and GEC differentiation^52^. Additionally, the glomerular parietal epithelium secretes various cytokines, hormones, and bioactive substances, such as aldosterone, vasopressin, and prostaglandins, which can influence GEC function^56^. Simultaneously, studies have also found that GECs can regulate the survival, proliferation, and apoptosis of glomerular epithelial cells in the glomerular wall layer through the epidermal growth factor (EGF)/epidermal growth factor receptor (EGFR) signaling pathway^53^,^54^ **(Figure 1)**.

## 4. The factors contributing to endothelial injury in chronic kidney disease

### 4.1 Inflammation and oxidative stress

Chronic low-grade inflammation is common in CKD, triggered by unresolved kidney damage^57^. This involves activation of the innate immune system, including monocytes, macrophages, and granulocytes, leading to persistent inflammation and endothelial cell damage^58^,^59^. Damaged cells release damage-associated molecular patterns (DAMPs) and toxins, increasing toll-like receptor (TLR) and NALP3 inflammasome expression in endothelial cells, which activates NF-κB^60^. This amplifies inflammation and increases ROS production while reducing NO bioavailability^61^,^62^, further damaging endothelial cells. Key inflammatory markers include elevated cytokines such as interleukin (IL)-1, IL-6, IL-18, TNF-α, C-reactive protein (CRP), and pentraxin-3 (PTX3), as well as adhesion molecules like vascular cell adhesion molecule-1 (VCAM-1), intercellular adhesion molecule-1 (ICAM-1), and monocyte chemoattractant protein-1 (MCP-1), all of which promote endothelial damage^63^.

Chemokines also contribute to this damage. For instance, in kidney diseases such as crescentic glomerulonephritis and diabetic kidney disease, CX3CL1, produced by renal endothelial cells, interacts with CX3CR1 to mediate inflammation^64^. TNF-α, interleukin-1 beta (IL-1β), and lipopolysaccharides (LPS) stimulate CX3CL1 expression, though its role in CKD requires further research^65^. CCR6 is constitutively expressed in glomerular endothelial cells but decreases during glomerular inflammation, suggesting its level can indicate endothelial damage^66^.

Complement activation also plays a role in kidney diseases, with anaphylatoxins from complement activation contributing to CKD by activating neutrophil inflammation, indirectly damaging glomerular endothelial cells^67^,^68^.

### 4.2 Factors Associated with Hemodynamics

#### 4.2.1 Blood Pressure

Hypertension often affects the kidneys and can lead to CKD, exacerbated by the overactivation of the renin-angiotensin-aldosterone system (RAAS) and the sympathetic nervous system, causing sustained high blood flow and pressure^59^. Hypertension also contributes to cardiovascular disease by damaging endothelial cells^69^.

Studies in hypertensive rats have shown impaired vasodilation, with increased sensitivity to vasoconstrictors such as angiotensin II and endothelin, and reduced NO levels, leading to endothelial damage^70^-^72^. Circulating endothelial microparticles (EMPs) are elevated in hypertensive patients, impairing vascular function and serving as early biomarkers of endothelial dysfunction^73^. Hypertension also alters endothelial progenitor cell numbers, gene expression, and lifespan, contributing to oxidative stress^74^.

Research suggests a mutual influence between blood pressure and endothelial cells, potentially creating a "vicious cycle"^75^. For example, inhibiting nitric oxide synthase increases arterial pressure, indicating that endothelial damage affects blood pressure regulation^76^.

#### 4.2.2 Shear Stress

Vascular endothelial cells respond to shear stress, which regulates their function. Laminar shear stress (LSS) in straight arteries supports endothelial cell growth and prevents apoptosis^77^,^78^. In contrast, oscillatory shear stress (OSS) in artery branches and curves promotes endothelial dysfunction, increases oxidative stress, and triggers inflammation, thereby raising the risk of cardiovascular disease in CKD patients^79^.

OSS induces oxidative stress via NADPH oxidase, activating pro-inflammatory signals such as NF-κB and disrupting endothelial eNOS function^77^. Integrins interact with extracellular matrix proteins, activating RhoA and mitogen-activated protein kinases (MAPKs), which regulate endothelial cell proliferation, migration, and morphological changes^80^. This process leads to high cell turnover and replicative senescence, particularly at arterial bifurcations, contributing to atherosclerosis^81^,^82^.

Low shear stress may induce endothelial dysfunction through the liver kinase B1 (LKB1)/AMP-activated protein kinase (AMPK)/p47phox pathway. Studies have shown that glycocalyx shedding under OSS is associated with increased endothelial injury markers, indicating a potential pathway for endothelial damage^83^.

### 4.3 Factors Related to Metabolism

#### 4.3.1 Glycometabolism

Chronic hyperglycemia is the leading cause of diabetes-related renal microvascular complications. Metabolic dysregulation, increased ROS, activation of the polyol pathway, and the formation of advanced glycation end products (AGEs) contribute to early endothelial dysfunction^84^,^85^. Elevated glucose promotes oxidative stress in endothelial cells, reduces NO bioavailability, and inhibits sirtuin proteins and histone acetyltransferases, which suppress forkhead box O1 (FOXO1) activity and induce ROS generation^86^.

Studies have shown that endothelial cells rely heavily on anaerobic glycolysis for energy^87^. However, diabetes-induced endothelial dysfunction involves mitochondrial defects, leading to elevated ROS levels and further damage^88^. Hyperglycemia also reduces telomerase activity and endothelial eNOS phosphorylation, thereby lowering NO production^89^,^90^.

Hyperglycemia promotes vascular dysfunction by thinning the glycocalyx, thereby reducing its protective role^91^. Increased glycocalyx shedding and oxidative stress markers indicate impaired endothelial function^92^. Additionally, hyperglycemia induces an inflammatory environment, affecting ICAM, VEGF, and Notch signaling, ultimately leading to endothelial cell apoptosis and glycocalyx degradation^93^.

#### 4.3.2 Amino Acid Metabolism

Homocysteine, a methionine metabolite, is associated with endothelial damage^94^-^96^, particularly in advanced CKD patients with hyperhomocysteinemia^97^. Elevated homocysteine levels stimulate hydroxyl radical production, reduce NO activity, and increase oxidative stress, leading to endothelial dysfunction^98^,^99^. Studies have shown higher homocysteine levels in patients with coronary artery disease and endothelial dysfunction^100^. Homocysteine-mediated low-density lipoprotein (LDL) oxidation further damages the endothelium by altering mitochondrial gene expression and promoting oxidative stress^98^,^101^.

#### 4.3.3 Lipid Metabolism

CKD patients often experience lipoprotein metabolism disorders, characterized by abnormal lipid profiles and the accumulation of atherogenic particles^102^,^103^, which contribute to endothelial damage via oxidative stress and inflammation^104^,^105^.

High-density lipoprotein (HDL) normally protects against LDL oxidation by ROS; however, in CKD, HDL's protective functions are impaired due to decreased apolipoproteins and abnormal post-translational modifications^106^-^108^. CKD-related HDL dysfunction reduces eNOS activation and impairs endothelial repair. Moreover, paraoxonase 1 (PON1) deficiency in CKD further diminishes HDL's antioxidant capacity, exacerbating LDL oxidation and endothelial damage^34^,^103^,^109^,^110^.

#### ATP and Energy Uptake

Endothelial cell stability depends on energy metabolism, particularly ATP production. ATP generated by endothelial cell mitochondria regulates vascular tone by controlling calcium-dependent nitric oxide (NO)-mediated relaxation^111^. ATP deficiency or disruption of calcium influx can lead to endothelial dysfunction and proteinuria^112^-^114^. Studies have shown that ATP influences endothelial fenestrae stability, cytoskeleton maintenance, and cell connections. In CKD patients, decreased ATP levels result in impaired vascular tension control and endothelial barrier damage caused by prolonged ischemia and hypoxia^115^-^118^.

## 5. Outcomes of Endothelial Injury in CKD

### 5.1 Albuminuria

Patients with cardiovascular conditions, such as hypertension and heart failure, often exhibit trace albuminuria, which signals endothelial barrier damage, including glycocalyx injury and endothelial dysfunction^119^. The presence of albuminuria in cardiovascular diseases indicates shared pathophysiological processes, such as endothelial dysfunction, chronic inflammation, and increased vascular leakage^120^. A study by Stephen L. Seliger and colleagues confirmed a close association between microvascular endothelial dysfunction, significant albuminuria, and CKD, underscoring the systemic cardiovascular risk in these patients^121^.

Early research identified a correlation between the prevalence of microalbuminuria and the severity of hypertension^122^,^123^. Sparving and colleagues first described the association between primary hypertension and microalbuminuria in 1974, noting that urinary albumin excretion increased with blood pressure but decreased when blood pressure was controlled^124^. Microalbuminuria is also associated with glomerular endothelial glycocalyx damage. Studies on rat kidneys demonstrated that albumin remains confined to the glomerular capillary lumen, indicating that the endothelial surface regulates albumin leakage^125^. In vitro studies further revealed that removing the glycocalyx reduces endothelial resistance and increases albumin flux^126^.

In the early stages of diabetes, GEC dysfunction serves as an early marker of diabetic nephropathy. Elevated glucose levels induce mitochondrial dysfunction and increase ROS, which damage endothelial cells and the glomerular filtration barrier (GFB), leading to albuminuria^85^. In diabetic patients, increased endothelial cell surface adhesion molecules and selectins exacerbate injury. Research has shown that platelet activation via the mTORC1 pathway contributes to GEC damage^127^.

The GFB also functions as an electrical charge barrier that repels negatively charged proteins, preventing albumin leakage. Studies have demonstrated that glycocalyx thinning reduces the charge selectivity of the GFB, resulting in albuminuria^128^,^129^. Increased expression of proteinases, such as MMP9, hyaluronidase, and heparanase, in diabetic patients degrades the endothelial glycocalyx, compromising the charge barrier and exacerbating albuminuria^130^,^131^.

### 5.2 Edema

Edema, the accumulation of excess fluid in tissues, is traditionally attributed to inadequate blood volume and activation of the renin-angiotensin-aldosterone system. However, changes in the endothelial filtration barrier also contribute to edema development^132^. The low-filling theory proposes that proteinuria and hypoalbuminemia reduce serum osmotic pressure, resulting in edema. Research has shown that, in some patients, a primary renal defect in sodium and water excretion increases plasma volume, leading to overflow edema. Clinical studies have identified an increased capillary filtration coefficient (CFC) and elevated capillary permeability as key factors in peripheral edema^133^. Tight junctions between endothelial cells regulate hydraulic conductivity, and hypoalbuminemia may enhance capillary permeability by promoting intracellular calcium influx^134^.

### 5.3 Coagulation

CKD patients are at higher risk of coagulation disorders due to the loss of coagulation inhibitors through excretion and increased fibrinogen production^135^-^137^. Endothelial dysfunction contributes to venous thrombosis, with uremic toxins activating endothelial cells to exhibit procoagulant properties^138^. Elevated levels of the endothelial injury marker ProET-1 and depletion of platelet granules have been observed in end-stage CKD^139^. Inflammation-driven immune thrombosis further exacerbates fibrin formation and local clotting^140^.

Although CKD patients have an increased risk of venous thrombosis, they typically do not develop disseminated intravascular coagulation (DIC), as DIC is more commonly associated with acute illnesses, whereas CKD follows a chronic course^141^.

## 6. Improving CKD by Intervening in Endothelial Cells

### 6.1 Vascular Protective Factors

Vascular protective factors are critical in managing CKD, as they enhance vascular function, regulate blood pressure through vasodilation, and reduce inflammation and oxidative stress, thereby protecting the endothelium and maintaining vascular health in CKD patients. Among these factors, nitric oxide (NO) plays a pivotal role. Reduced NO bioavailability is a hallmark of CKD progression, particularly in end-stage kidney disease (ESKD). This reduction is driven by various factors, including the accumulation of endogenous eNOS inhibitors, oxidative stress, inflammation, AGEs, disturbances in bone mineral metabolism (e.g., hyperphosphatemia), elevated FGF23 levels, and deficiencies in active vitamin D and Klotho. Collectively, these factors contribute to endothelial dysfunction^142^,^143^.

Interventions aimed at increasing NO bioavailability have shown potential in improving endothelial function. For instance, the phosphate binder sevelamer has been shown to lower serum phosphate levels and enhance endothelium-dependent vasodilation in CKD stage 4 patients^144^,^145^. Similarly, vitamin D analogs, such as paricalcitol, have demonstrated therapeutic efficacy in preserving endothelial integrity. Research by Amanda Lima Deluque et al. found that paricalcitol treatment in ARD rats increased eNOS/NO expression, reduced oxidative stress, and inhibited the TGF-β1/Smad2/3 pathway, thereby restoring endothelial structure and function^146^-^148^.

Furthermore, endothelial cell factors like soluble fms-like tyrosine kinase-1 (sFlt-1/sVEGFR1), a natural antagonist of VEGF, play a complex role in CKD. While sFlt-1 helps regulate VEGF activity to prevent excessive angiogenesis, elevated circulating sFlt-1 levels have been associated with endothelial dysfunction in CKD patients and post-kidney transplantation. Heparin administration during hemodialysis can further increase sFlt-1 secretion, exacerbating endothelial damage. However, clinical evidence regarding the benefits of targeting sFlt-1 levels to improve kidney and cardiovascular outcomes remains insufficient^149^.

### Gene Therapy

Hypoxia-inducible factors (HIFs) regulate genes critical to the survival, metabolism, and angiogenic activity of vascular endothelial cells, playing a pivotal role in vascular development and diseases, including CKD^150^,^151^. Endothelial cell dysfunction is considered a key factor in the progression of AKI to CKD, with prolyl hydroxylases (PHD) 1-3 playing a crucial role in regulating kidney repair following ischemia^152^. Researchers developed a transgenic mouse model using Cdh5Cre (PAC)ER to induce the specific inactivation of PHD2 in endothelial cells, either alone or in combination with PHD1 and PHD3. Their findings highlight the multifaceted effects of the PHD/HIF pathway on vascular endothelial cells. Notably, metabolic alterations are associated with the upregulation of solute carrier family 16 member 3 (SLC16A3), which encodes monocarboxylate transporter 4 (MCT4). This regulation selectively impacts the endothelial cell hypoxia-driven glycolysis/MCT4 axis, effectively preventing the progression from AKI to CKD. Furthermore, the study demonstrated that MCT4 inhibition could attenuate the inflammatory activation of endothelial cells and reduce interactions between monocytes and endothelial cells. These findings suggest that both gene silencing and pharmacological inhibition of MCT4 hold potential as therapeutic strategies for reprogramming endothelial cell metabolism comprehensively^153^.

Despite the promising prospects of gene therapy, it faces several challenges. The high research and production costs, particularly for personalized gene editing technologies such as CRISPR-Cas9^154^, result in expensive treatments. Additionally, gene therapy requires customization based on patients' genetic characteristics, making the production process complex and difficult to scale up. Safety concerns are another significant issue, as gene editing may lead to off-target effects, causing unforeseen side effects such as cancer or other genetic disorders^155^. The use of viral vectors in gene therapy can also trigger immune reactions, leading to treatment failure or severe side effects^156^. Therefore, the long-term effects and potential risks of gene therapy require further investigation, particularly concerning possible complications following gene editing.

### 6.3 Anti-Inflammatory and Antioxidant Therapy

In CKD patients, inflammation markers such as C-reactive protein and cytokines play a pivotal role in endothelial dysfunction and serve as independent predictors of CKD prognosis^157^. Targeting inflammation presents a promising strategy for protecting endothelial cells. The interplay between inflammation and oxidative stress is profound, with NF-κB activation and Nrf2 imbalance contributing to endothelial dysfunction. Notably, IL-6, regulated via the NF-κB pathway, is a critical biomarker for CKD prognosis^158^.

Patients with CKD and concurrent cardiovascular disease often exhibit abnormal lipid profiles, which exacerbate oxidative stress and inflammation. Statins, such as rosuvastatin, have been shown to significantly reduce CRP levels and lower cardiovascular event rates in CKD patients^159^. Omega-3 fatty acids may enhance endothelial health by increasing NO bioavailability, though large-scale clinical trials are still needed to confirm their efficacy^142^,^160^. Similarly, vitamin C, recognized for its anti-inflammatory properties, has demonstrated benefits in small-scale studies, including improvements in carotid intima-media thickness and flow-mediated dilation in CKD patients^161^.

Nonetheless, prolonged use of anti-inflammatory agents in CKD patients can increase infection risks and potentially worsen renal function, as observed with NSAIDs^162^,^163^. The variability in CKD progression, influenced by genetic factors and disease stage, complicates treatment decisions. This underscores the importance of precision medicine approaches, such as genetic testing and biomarker analysis, to optimize therapeutic strategies^164^. While anti-inflammatory treatments show short-term benefits, their long-term impact on CKD progression remains uncertain, necessitating further investigation^165^,^166^.

### 6.4 Blood Pressure Therapy

Multiple antihypertensive drugs, including ARBs, CCBs, and ACE inhibitors, can reverse endothelial damage in primary hypertension by modulating redox states and Ang-II receptor signaling^167^. Recent studies have also highlighted the role of the vasoconstrictor ET-1 in CKD-related endothelial damage^142^.

Amlodipine, an LTCC blocker, has been shown to slightly improve renal function and reverse endothelial dysfunction, likely through enhanced kinin activity, NO generation, antioxidant effects, and free radical scavenging^168^-^170^. Elevated ET-1 levels in CKD patients contribute to kidney injury via ETAR activation, which reduces NO production, increases oxidative stress, and promotes inflammation^171^. ETAR antagonists, such as zibotentan, have demonstrated efficacy in improving renal blood flow, reducing proteinuria, and ameliorating NO-mediated endothelial function^172^. They may also improve coronary atherosclerosis, a common CKD complication, though more research is needed on their effects in this population^173^,^174^.

Angiotensin II, similar to ET-1, causes endothelial damage by activating inflammatory pathways such as NF-κB and oxidative stress. ACE inhibitors, such as ramipril, have been shown to improve endothelial function (e.g., increased FMD) and reduce FGF-23 levels, a key contributor to endothelial dysfunction in CKD^175^-^177^. Further studies are needed to explore the long-term benefits of these therapies in CKD patients.

### 6.5 Blood Sugar-Lowering Therapy

Type 2 diabetes (T2D) often leads to microvascular complications, including CKD and ESRD^178^. Several antidiabetic medications, such as insulin, metformin, SGLT2 inhibitors, GLP-1 receptor agonists, and DPP-4 inhibitors, have shown protective effects on vascular endothelium by reducing oxidative stress and inflammation.

SGLT2 inhibitors consistently lower cardiovascular and renal event risks in T2D patients. For instance, empagliflozin improved endothelium-dependent vasodilation and reduced oxidative stress in diabetic mice after 8 weeks of treatment^179^,^180^. Similarly, the DEFENSE study demonstrated that dapagliflozin enhances endothelial function and glycemic control by reducing endothelial activation^181^.

DPP-4 inhibitors, especially when combined with insulin or metformin, also improve endothelial dysfunction in diabetic kidney disease (DKD). Linagliptin, for example, regulates endothelial markers like PECAM1, VEGF-A, and NOS3 by mitigating oxidative stress, as shown in a study by Hasan B Awal et al.^182^.

### 6.6 Lipid-Lowering Therapy

Statin-based lipid-lowering therapy has been shown to reduce proteinuria and slow renal function decline in CKD. The National Kidney Foundation recommends that CKD patients with LDL levels ≥100 mg/dL (2.59 mmol/L) should be managed with diet modifications or statins^183^. Statins can lower inflammatory markers, such as high-sensitivity C-reactive protein (HS-CRP), and improve endothelial function in high-risk cardiovascular populations^184^. Elevated total cholesterol and reduced HDL cholesterol are associated with an increased risk of CKD, and CKD patients face a higher risk of cardiovascular disease and mortality^185^,^186^.

Clinical studies indicate that atorvastatin improves endothelial function more effectively than ezetimibe, likely by reducing oxidative stress and upregulating eNOS^187^. Statins may also inhibit endothelial-to-mesenchymal transition (EndoMT). For example, lovastatin has been shown to protect endothelial cells in diabetic nephropathy by reducing oxidative stress and TGF-β1 signaling^188^.

However, statins may not mitigate all forms of endothelial injury. For instance, indoxyl sulfate (IS), a uremic toxin, increases endothelial activation markers (e.g., ICAM-1, VCAM-1), and atorvastatin does not significantly counteract IS-induced damage^189^. Thus, the role of statins in improving endothelial function in CKD requires further investigation.

### 6.7 Lifestyle Intervention

**Figure 2:**
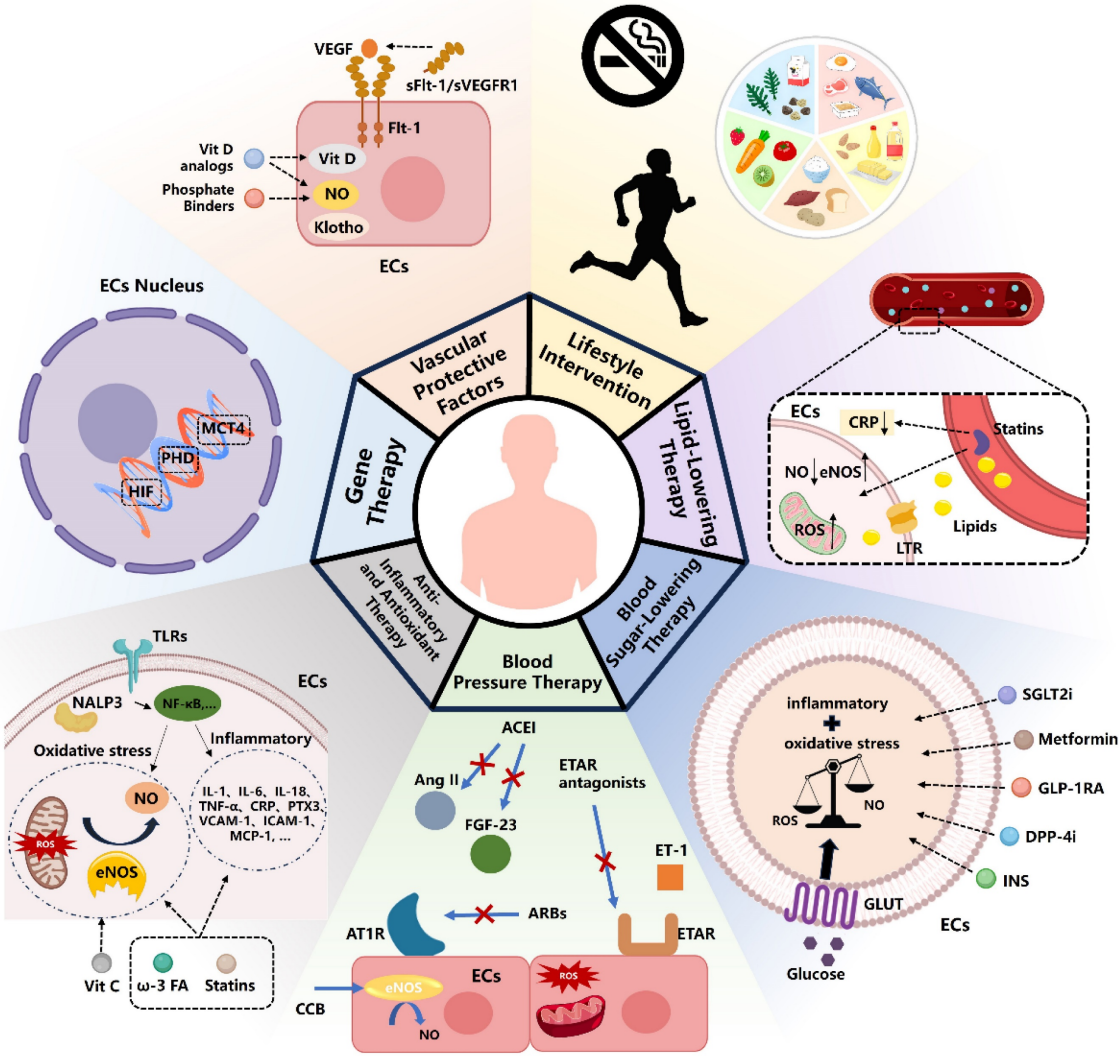
Treatment Strategies for Endothelial Injury in CKD Currently. ECs, Endothelial Cells; Vit D, Vitamin D; Flt-1, Fms-like tyrosine kinase 1; sFlt-1, soluble Fms-like tyrosine kinase-1; VEGF, Vascular Endothelial Growth Factor; ROS, Reactive Oxygen Species; eNOS, Endothelial Nitric Oxide Synthase; ω-3 FA, omega-3 fatty acids; Vit C, Vitamin C; ACEI, Angiotensin-Converting Enzyme Inhibitors; ARB, Angiotensin II Receptor Blockers; CCB, Calcium Channel Blockers; AT1R, Angiotensin II Type 1 Receptor; ET-1, Endothelin-1; ETAR, Endothelin A Receptor; FGF-23, Fibroblast Growth Factor 23; GLUT, Glucose Transporter; INS, Insulin; DPP-4i, Dipeptidyl peptidase 4 inhibitors; GLP-1RA, Glucagon-like peptide-1 receptor agonists; SGLT2i, Sodium-Glucose Co-Transporter-2 Inhibitors; LTR, lipid transport receptor; CRP, C-reactive protein.

Controlling blood pressure and blood sugar, as well as lifestyle changes such as maintaining a healthy weight and quitting smoking, can significantly improve endothelial health in CKD patients. Moderate exercise and dietary adjustments also play a crucial role in slowing the progression of the disease (**Figure 2**).

## 7. Conclusion

**Figure 3:**
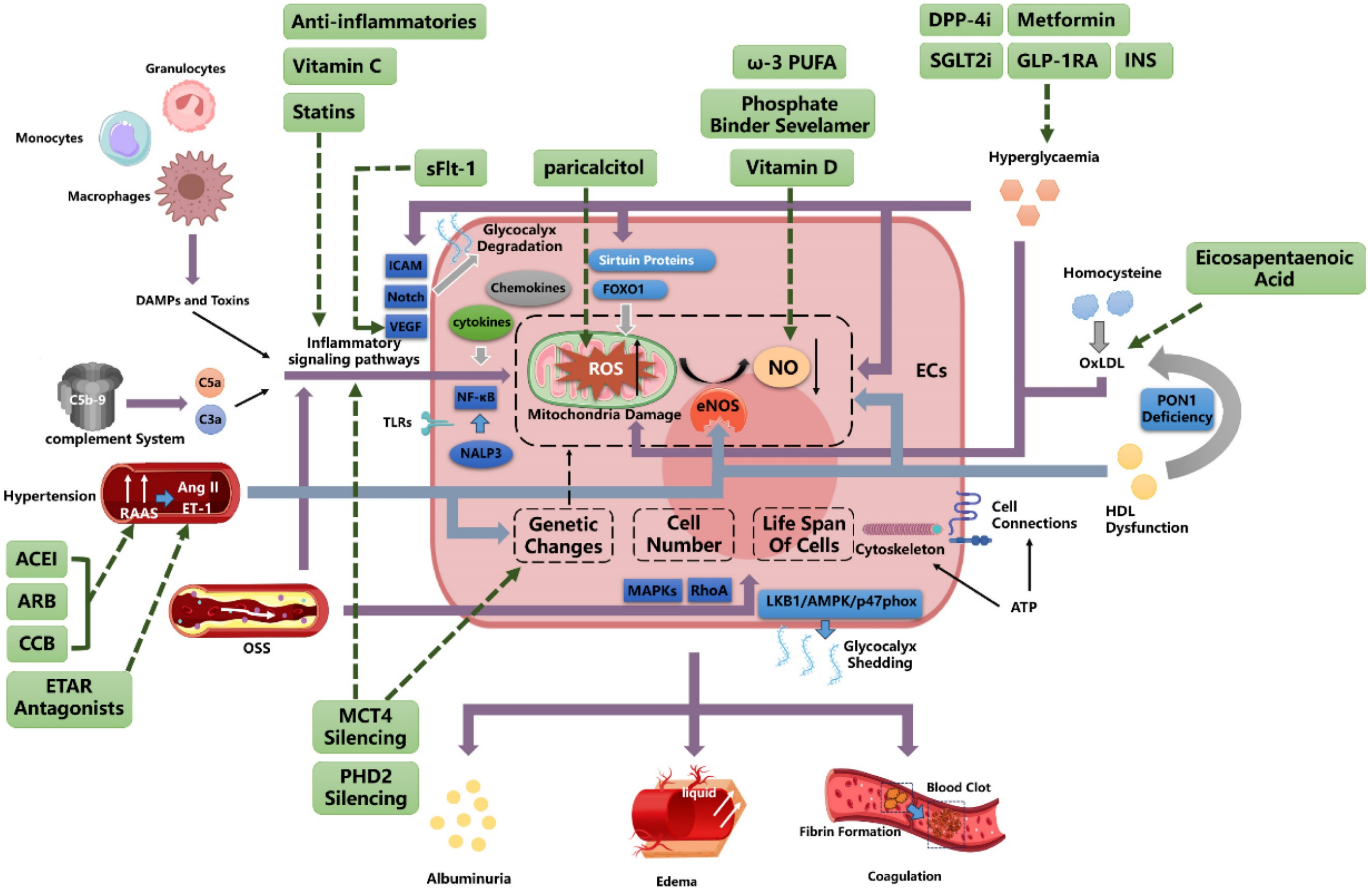
Integrated Mechanistic Network: Endothelial Dysregulation Pathways Converging with Cellular Interactions to Inform Therapeutic Targeting AECI, Angiotensin-Converting Enzyme Inhibitor; ARB, Angiotensin Receptor Blocker; CCB, Calcium Channel Blockers; ETAR, Endothelin A Receptor; ET-1, Endothelin-1; OxLDL, oxidized low-density lipoprotein; GLUT, Glucose Transporter; INS, Insulin; DPP-4i, Dipeptidyl peptidase 4 inhibitors; GLP-1RA, Glucagon-like peptide-1 receptor agonists; SGLT2i, Sodium-Glucose Co-Transporter-2 Inhibitors; sFlt-1, soluble Fms-like tyrosine kinase-1; VEGF, Vascular Endothelial Growth Factor; ROS, Reactive Oxygen Species; eNOS, Endothelial Nitric Oxide Synthase; ω-3 PUFA, omega-3 polyunsaturated fatty acid; ECs, Endothelial Cells.

Endothelial cell behavior in CKD is influenced by the internal environment, including inflammatory mediators and intercellular signaling pathways. The microenvironment regulates endothelial transcription factors and cell differentiation, leading to endothelial heterogeneity. This diversity contributes to the complex pathogenesis of CKD (**Figure 3**). Understanding the factors that drive endothelial dysfunction and heterogeneity is essential for developing new therapeutic strategies.

## Supplementary Material

Supplementary table.

## Figures and Tables

**Table 1:** Summary of interaction of glomerular endothelial cell (GECs) with surrounding cells under physiological conditions

Interaction with GECs	Mediators	Related Pathways or Mechanisms	Physiological effects	Reference(s)
Podocyte → GECs	VEGF-A	VEGF-A/VEGFR2 signaling pathway	Promoting endothelial cell differentiation and development, maintaining endothelial cell structure and function	^37^
	VEGF-C	VEGF-C/VEGFR3 signaling pathway	Increasing fenestration density in endothelial cells, reducing albumin permeability	^36,38^
	Ang-1	Ang-1/Tie-2 signaling pathway	Maintaining vascular integrity, enhances cell survival, promotes vascular stability, and facilitating angiogenesis; Inhibiting injury-induced angiogenesis and fibrosis	^39^
	ET-1	ET-1 signaling pathway	Promoting endothelial cell proliferation, enhances cytokine production, and affecting the regulation of inflammatory responses and fibrosis in glomerular endothelial cells	^40^
GECs→ podocyte	VEGF-A	VEGF-A/VEGFR1 signaling pathway	Protecting podocytes from apoptosis by promoting nephrin phosphorylation and enhancing podocin-CD2AP interaction	^36,37^
	ET-1	ET-1/ETAR signaling pathway	Influencing the morphology, function, and proliferation of podocytes; Regulating the synthesis and secretion of extracellular matrix proteins by podocytes, influencing podocyte adhesion, migration, and invasion	^41^
TECs→ GECs	Ang-(1-7)	Ang-(1-7)/Mas signaling pathway	Leading to sustained activation of the klotho and Nrf2/HO-1 signaling pathways, collectively inhibiting the aging process of GECs	^42,43^
	VEGF-A	VEGF-A/ VEGFR2 signaling pathway	Promoting endothelial cell differentiation and development, maintaining endothelial cell structure and function	^37,44^
	VEGF-C	VEGF-C/VEGFR3 signaling pathway	Increasing fenestration density in endothelial cells, reducing albumin permeability	^36,38^
GECs→ TECs	IGFBPs	IGF signaling pathway	Producing IGFBP4, IGFBP-2, and IGFBP-3, and express mRNA for IGFBP-2 to IGFBP-5, regulating IGF signaling in TECs and influencing renal tubular function	^45^
MCs→ GECs	Ang-2	Ang-2/Tie-2 signaling pathway	Regulating endothelial cell proliferation	^43,46^
	VEGF-A	VEGF-A/VEGFR2 signaling pathway	Inhibiting Tie2 phosphorylation and promoting endothelial cell proliferation	^47,48^
GECs→ MCs	PDGF-B	PDGF-B/PDGFR-β signaling pathways	Promoting the differentiation and development of MCs	^49^
	NO	Nitric oxide-mediated signaling pathways	Stimulating cGMP production in MCs through a NO-dependent pathway	^50^
	Exosome containing TGF-β1 mRNA	TGFβ1/Smad3 signaling pathways	Promoting cellular proliferation and extra cellular matrix production	^46,51^
PECs→ GECs	VEGF-A	VEGF-A/VEGFR2	Inhibiting Tie2 phosphorylation and promoting endothelial cell proliferation	^52^
GECs→ PECs	EGF	EGF/EGFR	Regulating cell survival, proliferation and apoptosis	^53,54^

GECs: Glomerular Endothelial Cells; TECs: Tubular Epithelial Cells; MCs: Mesangial Cells; PECs: Parietal Epithelial Cells; VEGF: Vascular Endothelial Growth Factor; VEGFR: Vascular Endothelial Growth Factor Receptor; Ang-1: Angiopoietin-1; ET-1: Endothelin-1; ETAR: Endothelin A Receptor; IGFBPs: Insulin-like Growth Factor Binding Proteins; NO: Nitric Oxide; PDGF-B: platelet-derived growth factor-B; TGF-β1: Transforming growth factor Beta 1; EGF: Epidermal Growth Factor; EGFR: Epidermal Growth Factor Receptor; cGMP: cyclic guanosine monophosphate
