# Supplementary material for: Heterogeneity of Renal Endothelial Cells, Interact with Neighboring Cells, and Endothelial Injury in Chronic Kidney Disease: Mechanisms and Therapeutic Implications
Source: Int J Med Sci. 2025 Apr 9;22(9):2103–18. doi: 10.7150/ijms.108299 (PMC12035827; doi:10.7150/ijms.108299)
Supplement: Supplementary file 1 — Supplementary table. [file ijmsv22p2103s1.pdf]

Supplementary Material

Table S1. Genes and Functions of Subtypes of Renal Endothelial Cells

**Renal Endothelium**	**Subtype**	**Genes**	**Known functions**
**cortical endothelium**	Afferent arteriolar ECs	*Bmp4, Alox12, Slc18a1, Edn1, Gja4, S1pr1, Cldn5, Cxcl12, Kcnn4*	Regulating angiotensin and renal blood flow
	Capillary ECs with high ApoE expression	High *ApoE, Plpp3 ,Thrsp*	Related to lipid metabolism
	Capillary ECs with low ApoE expression	Low *ApoE*	Regulating blood volume and sodium excretion
	Angiogenic capillary ECs	*Apln, Aplnr, Col4a1, Col4a2, Esm1, Fscn1, Gpihbp1, Plk2, Trp53i11*	Involved in the regeneration of damaged ECs
	Interferon-activated ECs	*Isg15* and *Ifit family genes*	Exhibit an IFN-response phenotype;<br>Involved in immune surveillance
	Post-capillary	*Jup, Nr2f2, Tnxb, Kdr*	Drives the transcriptional tagging and identification of specific vascular beds;<br>Maintaining renal tubular reabsorption function
	Efferent arteriolar ECs	*Klf4, Calca, Cryab, Slc6a6*	Regulating calcium influx.
	Large artery ECs	*Mgp, Eln, Gja5, Slc8a1, Kcnn4, Cldn5, Ltbp4, Bmp4, Ace, Cldn5, Jag1, Edn1*	Related to elastin fiber assembly; Regulating angiotensin
	Large vein ECs	*Plvap, Gas6, CD9, Nr2f2*	Facilitating the exchange of water, ions, and small solutes with the proximal and distal renal tubules;<br>Involving in endothelial fenestration
**medullary endothelium**	Microvascular	*Fbln5, Gja4, Sox17*	Urine concentration
	Descending vasa recta ECs	*Crip1, Slc14a1, Scin, Aqp1, Cldn5, Hpgd, End1, Cxcl12, Adipor2, and Slc5a3*	Facilitating the transport of aquaporin-2; Involved in the metabolism of vasodilatory prostaglandins

	Papillary descending vasa recta ECs	*Nrgn, S100a4, S100a6, Fxyd5, Akr1b3*	Concentrating urine; maintaining cell volume under high osmotic conditions.
	Fenestrated capillary ECs	*Plpp3, Cd36*	Fatty acid transport and metabolism
	Angiogenic capillary ECs	*Fscn1, Col4a1, Esm1, Col4a2, Trp53i11, Aplnr, Apln, Plk2, Gpihbp1*	Promotes the development of renal vasculature and podocytes
	Interferon-activated capillary ECs	*Isg15* and *Ifit family genes*	Immune-related
	Post-capillary venule ECs	*Jup, Nr2f2, Tnxb, Kdr*	Maintaining water reabsorption in renal tubules
	Ascending vasa recta ECs	*Fxyd6, Gas6, Tek*	Urine concentration
	Papillary ascending vasa recta ECs	*Ldha, Gapdh, Crip1, Nrgn, Aldoa, Akr1b3, Fxyd5, S100a6*	Urine concentration and glycolysis
	Interferon-activated vein ECs	*Isg15* and *Ifit family genes*	Immune-related
**glomerular endothelium**	Afferent arteriolar ECs	*S1pr1, Gja4, Alox12, Kcnn4, Edn1*	Involved in the regulation of angiotensin
	Afferent arteriolar/JGA ECs	*Cd300lg, Gja5*	Regulating renin release and blood pressure
	Efferent arteriolar ECs	*Cryab, Klf4, Calca, Slc6a6*	Involved in osmotic response
	Efferent arteriolar/JGA ECs	*Gas5, Ptprr, Slc26a10*	Involved in immune cell adhesion and extravasation; Regulating endothelial cell permeability
	Glomerular capillary ECs	*Scn7a, Sema5a, Nostrin, Abcc4, Ehd3, Lpl, Smad6*	Maintaining fenestration of glomerular capillaries; Promoting the formation of capillaries

ECs endothelial cells; IFN Immune Interferon
